# Pharmacological and Non-Pharmacological Interventions for Polycystic Ovary Syndrome (PCOS) in Indian Women: A Systematic Review and Meta-Analysis

**DOI:** 10.3390/ph18050680

**Published:** 2025-05-02

**Authors:** Pratibha Maan, Rohit Gautam, Sudharsan Vasudevan, Geetha R. Menon, Amit Arora, Abilash Nair, Puthiyaveettil Khadar Jabbar, Taruna Arora

**Affiliations:** 1 Indian Council of Medical Research (ICMR) Headquarters, New Delhi 110029, India; maanpratibha@gmail.com (P.M.); rohitgautam692@gmail.com (R.G.); dr.sudharsan.pcos@gmail.com (S.V.); 2 Department of Health Research, Ministry of Health and Family Welfare, Government of India, New Delhi 110001, India; menongr.hq@icmr.gov.in; 3 Sir Ganga Ram Hospital, New Delhi 110060, India; amitsgrh@yahoo.com; 4 Department of Endocrinology, Government Medical College, Thiruvananthapuram 695011, India

**Keywords:** polycystic ovarian syndrome, insulin sensitizer, metformin, anti-androgens, anti-obesity drug, ovulation induction drugs

## Abstract

**Background:** Polycystic ovary syndrome (PCOS) is an endocrine disorder prevalent in women of reproductive age. Treatment or management of this syndrome includes several pharmacological and non-pharmacological treatment approaches for different manifestations of the disease that vary with the patient’s age, symptoms, requirements, and geographical location. **Objective:** This systematic review aims to conduct a comprehensive and evidence-based analysis of the various available treatment options and identify knowledge gaps in PCOS management in India. **Methods:** A comprehensive search was conducted in PubMed, Scopus, and Embase databases from January 2010 till February 2024. We included randomized control trials (RCTs) using any pharmacological drugs (e.g., insulin sensitizers, anti-androgens, anti-obesity drugs, oral contraceptive pills, ovulation induction drugs, etc.) or non-pharmacological intervention (e.g., yoga, diet, herbal supplements, etc.) with Indian PCOS patients for improving common manifestations of PCOS and written in the English language. Studies were screened by two authors independently in a two-level process. Data extraction was also performed by two authors. Risk of bias was performed using the RoB 2 Tool. Subgroup analysis and meta-analysis were performed using the RevMan tool. **Results:** Thirty RCTs on pharmacological and eight on non-pharmacological interventions were included in the study. However, all the RCTs were so heterogeneous in terms of intervention used, subject recruited, and outcomes measured that meta-analysis was possible for only three subgroups (metformin vs. inositol, metformin vs. metformin+ inositol, and letrozole vs. clomiphene citrate), with only two or three studies per analysis. Most studies were single-centric and small-sized and had a high risk of bias, limiting their generalizability. **Conclusions:** This systematic review synthesized existing research and evaluated the effectiveness and safety of existing treatments. Limitations and gaps in the current research were identified, which may inform future research for better understanding and management of PCOS in the Indian context.

## 1. Introduction

### 1.1. Polycystic Ovarian Syndrome (PCOS)

PCOS, a complex reproductive endocrine disorder, poses a significant threat to the health of women in adolescence and reproductive age [1]. Its prevalence varies from 4% to 20% worldwide [2]. According to Bharali et al. (2022), approximately 10% of women, as per the Rotterdam criteria, and 5.8%, as per the National Institutes of Health (NIH) criteria, are affected by PCOS in India [3]. Primary symptoms of this syndrome include chronic ovulatory anovulation (such as oligomenorrhea, amenorrhea, and menstrual irregularities), hyperandrogenism (leading to acne, hirsutism, etc.), multiple cysts in ovaries, and other associated comorbidities like obesity, dyslipidemia, diabetes mellitus, and hypertension leading to cardiac dysfunction over a long period [4]. Oligomenorrhea, hirsutism, and acne in adolescent-age women and infertility in reproductive-age married women are the primary manifestations that have a devastating impact on those affected. PCOS may also affect the psychosocial well-being of patients, as it leads to stress, depression, anxiety, reduced self-esteem, eating disorders, and a decrease in the overall quality of life [5,6]. Various factors like the lifestyle of the individual, exposure to environmental pollutants, and genetic and epigenetic factors are responsible for the etiology of PCOS [7]. Diagnosis is mainly based on the presence of three conditions: menstrual cycle disruption, clinical and/or biochemical hyperandrogenism, and the presence of multiple cysts in the ovary in the ultrasound image [8]. There are three distinct criteria available for diagnosing PCOS—the NIH, Rotterdam 2003, and Androgen Excess Society criteria [9,10,11]. The Rotterdam criteria are usually more liberal as they require the presence of any two symptoms out of three and diagnose more women as having PCOS as compared to the NIH criteria [8]. Currently, the Rotterdam criteria are the most preferred and accepted criteria for diagnosing PCOS [12].

### 1.2. Current Treatment Options

Currently, there is no complete cure for PCOS; treatment focuses on symptom relief and preventing long-term consequences such as endometrial cancer and cardiovascular problems and enhancing the quality of life [13]. Management of PCOS is multidisciplinary. The first line of management of the condition is lifestyle modification strategies like calorie-restricted diet, exercise, and behavioral interventions targeted towards weight reduction, which also helps with insulin resistance, especially among obese individuals. However, there is a lack of clear guidelines on specific types of physical activity and diet to be followed. Metformin therapy combined with conservative management or even bariatric surgery has been recommended for weight reduction and management of insulin resistance and hyperandrogenism, especially in overweight/obese women [12,14,15]. For managing menstrual irregularity and hormonal imbalance, oral contraceptives seem to be recommended the most, while metformin, cyproterone acetate, and drospirenone are some of the other commonly used drugs [15,16]. Various treatments for hirsutism and acne have been suggested, including photoepilation, topical eflornithine, anti-androgen medications, and hormonal contraceptives (Legro et al., 2013 [17]; Teede et al., 2018 [12]). In addressing infertility, lifestyle treatments are considered the optimal first line for anovulation, followed by pharmacological ovulation induction using letrozole, but it is not recommended for women who are overweight [18]. Gonadotropins or laparoscopic ovarian drilling have also been recommended as alternative treatment options, with in vitro fertilization as a last resort [9,14,19]. However, various existing treatments have adverse effects. For instance, the use of oral contraceptives (OCPs) is linked to weight gain [20]. Metformin usage is associated with gastrointestinal (GI) problems [21], and fertility treatments pose an increased risk of ovarian hyperstimulation syndrome (OHSS) [22].

In summary, due to its diverse nature, a one-size-fits-all approach to treatment is often ineffective. The approach for managing PCOS is highly individualized and planned to each patient’s specific symptoms and needs. Health professionals have to thoroughly assess the patient and decide the treatment strategy based on each individual’s unique circumstances.

### 1.3. Rationale for Systematic Review

Currently, India lacks tailored treatment guidelines for PCOS, and existing international guidelines do not account for the country’s diverse ethnicity, genetics, lifestyle variations, environmental factors, and limited primary care infrastructure. This gap leads to inconsistent and less-effective management of PCOS in the Indian population. Therefore, there is need for evidence-based approaches to improve the effectiveness and consistency of PCOS management in India. This systematic review aims to determine the various interventions being used for PCOS and their effectiveness in the Indian population, to develop evidence-based, region-specific guidelines for better healthcare outcomes.

## 2. Objectives of Review

In order to bring an evidence-based approach towards the management of PCOS, we aim to conduct this systematic review for the following reasons:

1. To determine different pharmacological interventions available and tested for the management of different manifestations of PCOS among Indian women;
2. To determine different non-pharmacological intervention approaches tested for the management of different manifestations of PCOS among Indian women;
3. To identify knowledge gaps in PCOS management in India.

## 3. Methods

### 3.1. Study Design and Registration

This systematic review complies with the standard of PRISMA-P2020 (Preferred Reporting Item for Systematic Review and Meta-analysis) guidelines [23]. The protocol has been registered with “The International Prospective Register of Systematic Reviews (PROSPERO)” with the ID CRD42024508759 [24].

### 3.2. Data Sources and Search Strategy

A comprehensive literature search was conducted through three electronic bibliographic databases: Pubmed, Embase, and Scopus, from 1 January 2010 to 1 March 2024. The following keywords along with their MesH (Medical Subject Headings) terms were used for the search: Population (PCOS OR Polycystic Ovarian Syndrome OR Polycystic Ovary Syndrome OR Polycystic Ovary Disease OR Polycystic Ovary Disorder OR PCOD); Intervention (Management OR Treatment OR Therapy OR Drugs OR Diet OR Exercise OR Herbal OR Surgery OR Lifestyle OR Novel Therapy OR Intervention Disease Managements OR Patient Care OR Therapeutic OR Therapies OR Treatments OR Medicine OR Physical Activity OR Yoga OR Plant Extracts OR Operative Procedures OR Surgical Procedure OR operative therapy OR Invasive procedures OR Remedy OR Insulin sensitizer OR Thiazolidinedione OR Biguanide OR Pioglitazone OR Metformin OR Liraglutide OR Sitagliptin OR Empagliflozin OR Atorvastatin OR Imeglemin OR Rimonabant OR Orlistat OR Sibutramine OR Glucagon receptor antagonist OR Naltrexone OR bupropion OR Sodium-glucose co-transporter-2 inhibitors OR Dipeptidyl peptidase-4 inhibitors OR Glucagon-like peptide-1 receptor analogue OR SGLT2 inhibitors OR Statins OR Oxidative OR Phosphorylation OR Myo-inositol OR Inositol OR clomiphene citrate OR letrozole OR Gonadotrophins OR In vitro fertilization OR IVF); and Study Design (Randomized control trial OR RCT OR Controlled clinical trial OR Non randomized controlled trial OR Clinical trial OR Single arm trial OR Quasi experimental study OR Cross sectional study). The details of the search strategy and retrieved results are attached as Supplementary File S1.

### 3.3. Inclusion/Exclusion Criteria for Studies

The eligibility criteria for selecting studies for inclusion in this systematic review are formulated according to the PIOS (Population, Intervention, Outcome, and Study Design) approach.

*Population*: Our population of interest was Indian women who have attained menarche and are diagnosed with PCOS.

*Intervention*: We included all the current and emerging treatment strategies used for PCOS in India. We included studies that evaluated the efficacy of pharmacological interventions (such as insulin sensitizers, anti-androgens, oral contraceptive pills, ovulation induction drugs, myo-inositol, weight loss medication, etc.) as well as non-pharmacological interventions (lifestyle modification, dietary modification, physical activity, exercise, yoga, supplementation, probiotics, herbal remedies, etc.).

*Outcome*: Our primary outcome was improvement in any of the common manifestations of PCOS, such as hyperandrogenism (acne, alopecia, hirsutism), menstrual irregularities (number of cycles/year), and hormone levels (levels of luteinizing hormone (LH), follicle-stimulating hormone (FSH), androgen, dehydroepiandrosterone sulfate (DHEAS), sex hormone-binding globulin (SHBG), etc.). Our secondary outcome was the treatment of other co-morbidities, such as infertility (pregnancy and live-birth rate) and metabolic syndrome (body mass index (BMI), blood pressure (BP), fasting blood glucose (FBS), triglycerides (TAGs), high-density lipoprotein (HDL), low-density lipoprotein (LDL), diabetes, etc.).

*Study design*: We included randomized control trials (RCTs), controlled clinical trials, non-randomized controlled trials, and prospective intervention studies conducted on Indian PCOS patients. Retrospective studies, single-arm studies, observational studies, case-control studies, cross-observational studies, systematic reviews, meta-analyses, review articles, descriptive studies, expert opinions, comments, and letters to editors were excluded from the study. Animal and in vitro studies were also excluded.

*Time duration:* The studies conducted from 1 January 2010 to 1 March were included.

*Language*: Studies written in English were included, and all other studies written in any other language were excluded.

*Availability of full text*: Studies whose full text was available were included, and all other studies were excluded.

### 3.4. Study Selection and Screening

The retrieved results were managed using RAYYAN’s blinded screening software (https://www.rayyan.ai/) [25]. Duplicate studies were removed. In preliminary screening, the title and abstracts of the studies were reviewed by two authors independently per the eligibility criteria. If there was any lack of agreement, the third reviewer resolved it. Then, the full-text articles and their potential references were downloaded and screened for final inclusion by two authors.

### 3.5. Data Extraction

Key findings regarding the pharmacological and non-pharmacological treatment available for managing PCOS in India were summarized in a descriptive synthesis of the included studies. The following data were collected in the characteristic table: citation, study design, criteria for PCOS, participant characteristics, location, intervention, sample size, duration of intervention, outcomes measured, and outcomes in which significant difference was observed between the groups. For studies included in the meta-analysis, data extraction was performed for all the outcomes measured. Three reviewers performed data extraction; each reviewer independently conducted a pilot test of the data extraction tool before its final implementation.

### 3.6. Assessment of Risk of Bias

All the included studies were appraised independently by two authors for the risk of bias in accordance with the revised Cochrane risk-of-bias tool for intervention studies (RoB 2). The assessment was conducted on six aspects of the study: sequence generation, allocation concealment, blinding of recruited patients and outcome assessors, follow-up of patients, reporting of the selective outcome, and other biases. Each study was categorized as “low-risk”, “high-risk”, or “unclear-risk”. The overall bias score for each study was designated based on the number of criteria of high risk it meets: “high risk of bias (>two)”, “moderate risk of bias (one to two)”, and “low risk of bias (zero)” [26].

### 3.7. Data Synthesis and Meta-Analysis

Initially, the findings from the included study were summarized narratively. The data permit meta-analysis for only three sub-groups, i.e., metformin vs. inositol, metformin vs. (metformin+ inositol), and letrozole vs. clomiphene citrate. The meta-analysis was conducted with the software Review Manager 5.3 (RevMan). The combined mean difference was used for continuous outcomes like blood glucose levels, fasting insulin, cholesterol, triglycerides, LDL, HDL, etc. Odds ratios (ORs) with 95% confidence intervals (95% CI) were reported as an overall synthesized measure of effect size. For visual assessment, a forest plot of summary was synthesized.

## 4. Results

### 4.1. Study Selection

**Figure 1:**
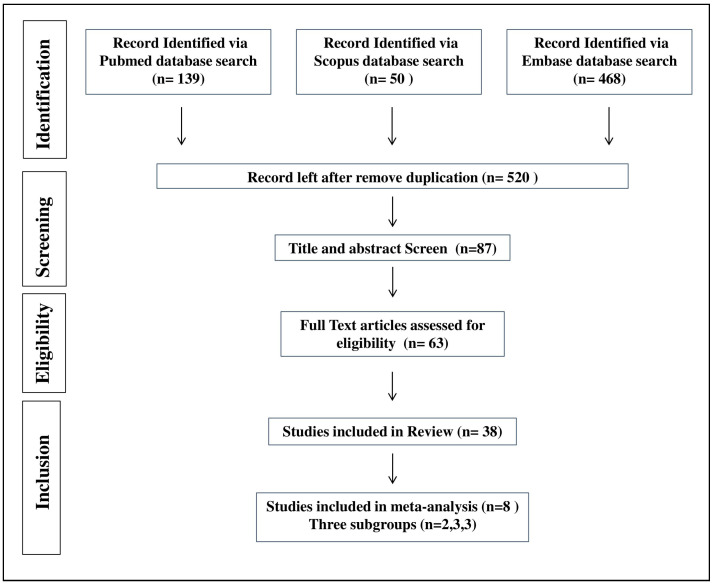
Preferred Reporting Item for Systematic Review and Meta-analysis (PRISMA) flow chart of study screening and selection process.

We initially retrieved 657 studies from Pubmed, Scopus, and Embase databases. After eliminating duplicates, 520 unique studies remained. Following primary screening based on title and abstract, 433 articles were excluded. Out of the remaining 87 articles, 16 were conference abstracts, and full texts could not be retrieved for eight studies even after contacting the authors. Subsequently, the full texts of the remaining 63 articles were assessed, and 38 studies were finally included in the systematic review. However, due to the diverse interventions used in these included studies, only eight studies were eligible for group and meta-analyses. The selection process is outlined in detail in Figure 1.

### 4.2. Description of Included Studies

Among the studies analyzed, there were 18 randomized controlled trials (RCTs), six open-label RCTs, five double-blinded RCTs, 1 assessor-masked RCT, 1 triple-blinded RCT, 1 non-randomized clinical trial, and six prospective intervention studies. Most of these studies have used the Rotterdam 2003 criteria for diagnosing PCOS. However, in four studies, Androgen Excess Society 2006 criteria were employed, two relied on physician discretion criteria, and three did not specify the diagnostic criteria used. The sample sizes of the included studies ranged from 15 to 60 in each group, with only two studies reaching a sample size of 100 participants/group. The distribution of studies conducted across various regions in India shows that Delhi had the highest number with seven studies, followed by Tamil Nadu with six.

### 4.3. Outcomes

Amongst the 38 included studies, we have subdivided the studies into pharmacological (*n* = 30) and non-pharmacological treatment (*n* = 8) groups. Pharmacological interventions were further divided into insulin sensitizers and anti-androgen drugs, anti-obesity drugs, oral contraceptive pills (OCPs), and ovulation induction drugs. Non-pharmacological interventions are classified into lifestyle modification (physical exercise and yoga), vitamin D (Vit D) supplement, probiotics, and herbal treatment. Since most of the RCTs have used a different type of intervention, in different combinations and doses, therefore, outcome-wise, meta-analysis was not feasible. Therefore, we have performed the intervention-wise meta-analysis for only three groups. For the rest of the RCTs, detailed characteristics and outcomes are mentioned in Table 1 and narrative synthesis is conducted in the next section. 

Amongst the 38 included studies, we have subdivided the studies into pharmacological (*n* = 30) and non-pharmacological treatment (*n* = 8) groups. Pharmacological interventions were further divided into insulin sensitizers and anti-androgen drugs, anti-obesity drugs, oral contraceptive pills (OCPs), and ovulation induction drugs. Non-pharmacological interventions are classified into lifestyle modification (physical exercise and yoga), vitamin D (Vit D) supplement, probiotics, and herbal treatment. Since most of the RCTs have used a different type of intervention, in different combinations and doses, therefore, outcome-wise, meta-analysis was not feasible. Therefore, we have performed the intervention-wise meta-analysis for only three groups. For the rest of the RCTs, detailed characteristics and outcomes are mentioned in Table 1 and narrative synthesis is conducted in the next section. 

Amongst the 38 included studies, metformin was part of 19 studies. Seven RCTs in our study compared the effect of metformin with inositol or their combination.

(a) Metformin vs. inositol

**Figure 2:**
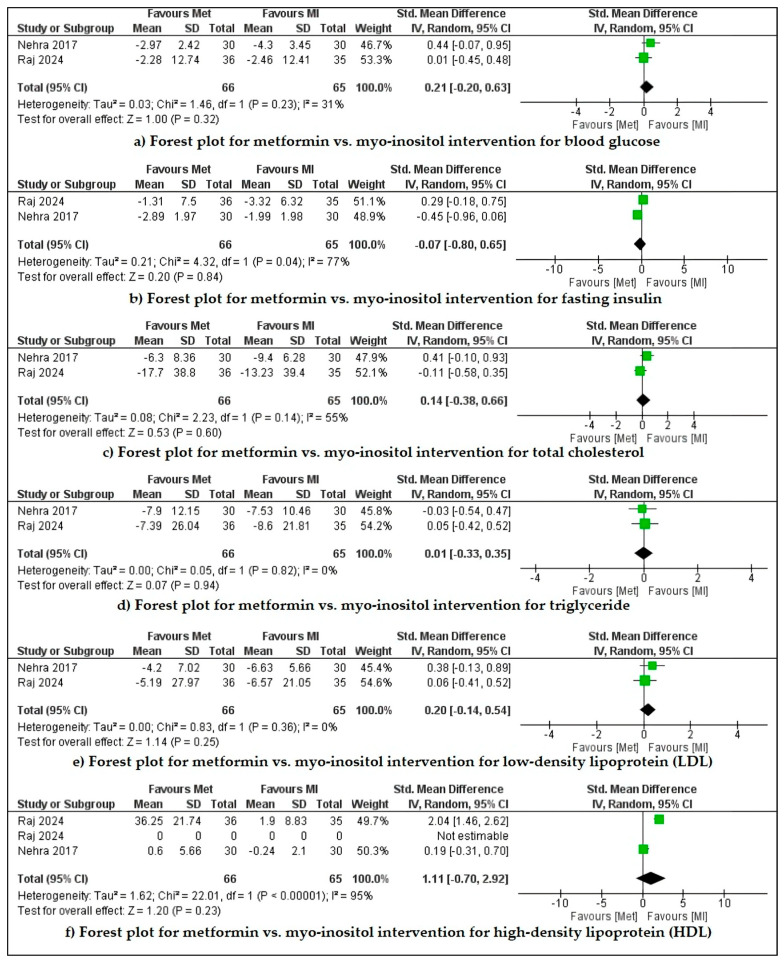
Forest plot for metformin vs. myo-inositol intervention for reducing blood glucose, fasting insulin, total cholesterol, triglycerides, and low-density lipoprotein (LDL) and increasing high-density lipoprotein (HDL) in Indian PCOS women.

Raj et al. (2024) and Nehra et al. (2017a) compared metformin with myo-inositol for menstrual cyclicity and biochemical and hormonal parameters [27,38]. These studies stated that both drugs efficiently improved clinical and biochemical outcomes, but intergroup differences are insignificant. Our meta-analysis revealed that metformin is slightly more effective for increasing HDL levels but is non-significant (*p* = 0.12) and has high heterogeneity (I^2^ = 94%, Cl 95%). For all other parameters, such as decreasing blood glucose, fasting insulin, cholesterol, triglyceride levels, and LDL level, intergroup differences are insignificant (*p* > 0.1) with very high heterogeneity (I^2^ = up to 77%, Cl 95%) (Figure 2). Insignificant improvement was observed in the hormonal profile in both of the cases. In the Raj et al. 2024 study, the metformin group showed a higher pregnancy rate than the myo-inositol group (50% vs 26.6%). Nehra et al. (2017b) compared metformin with myo-inositol for anthropometric parameters. The drugs reduce BMI, waist circumference, and hip circumference, but the intra-group and intergroup changes were insignificant [39].

(b) Metformin vs. combination of metformin and inositol

**Figure 3:**
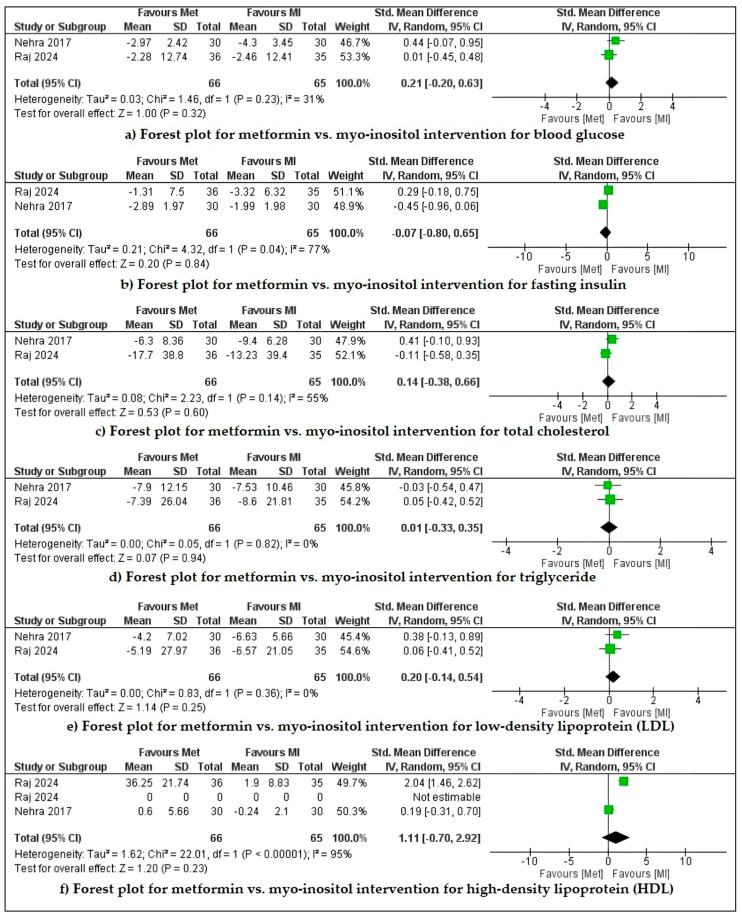
Forest plot for metformin vs. (metformin + myo-inositol) intervention for reducing BMI, fasting insulin, HOMA-IR, luteinizing hormone (LH), and serum testosterone in Indian PCOS women.

Three studies have compared metformin with the combination of metformin and inositol [28,30,35]. All the studies stated that both treatment plans could relieve clinical and biochemical symptoms. However, the meta-analysis revealed that intergroup differences are insignificant for all parameters examined such as BMI, fasting insulin, HOMA-IR, LH, and testosterone (Figure 3). Further, it is essential to mention that these three studies have used different concentrations of metformin, MI, and DCI (Table 1). Therefore, these studies have high clinical heterogeneity.

(c) Ovulation induction drugs

**Figure 4:**
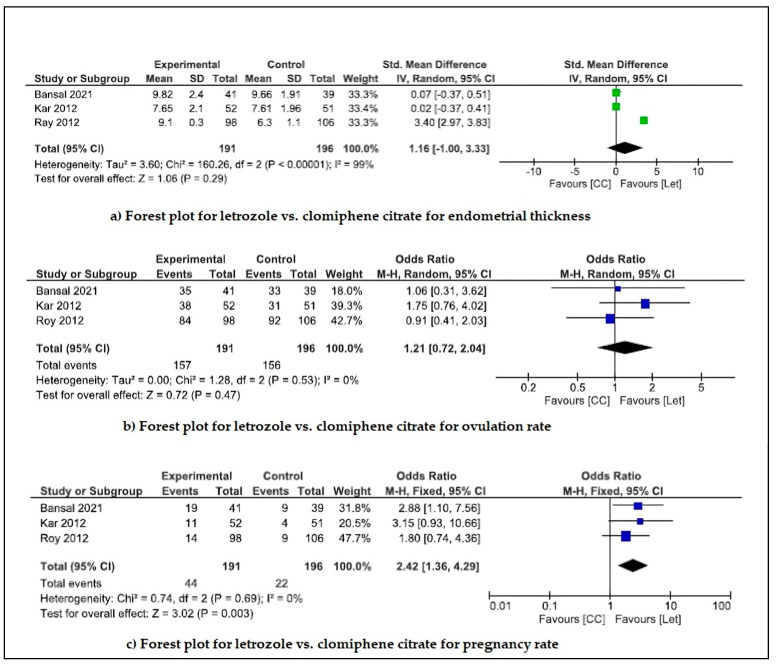
Forest plot for letrozole vs. clomiphene citrate interventions for reducing endometrial thickness and increasing ovulation and pregnancy rate in Indian PCOS women.

Letrozole and clomiphene citrate (CC) are the two most commonly prescribed drugs for ovulation induction in PCOS women with an infertility issue. In a study conducted by Kamath et al. (2010), it was found that letrozole can significantly increase the ovulation rate, follicular development rate, and progesterone as compared to the placebo in clomiphene citrate-resistance PCOS patients [56]. Three studies (Kar, 2012 [55]; Roy et al., 2012 [54]; Bansal et al., 2021 [52]) compared treatment with letrozole vs. CC for pregnancy rates, endometrial thickness, and ovulation rate (Figure 4). Meta-analysis revealed that letrozole is slightly more effective for increasing the pregnancy rate with a significant level (*p* = 0.003) and low heterogeneity (I^2^ = 0%, CI =95%). However, meta-analysis for parameters like endometrial thickness (*p* = 0.29) and ovulation rate (*p* = 0.47) showed no significant changes.

### 4.4. Risk of Bias

**Figure 5:**
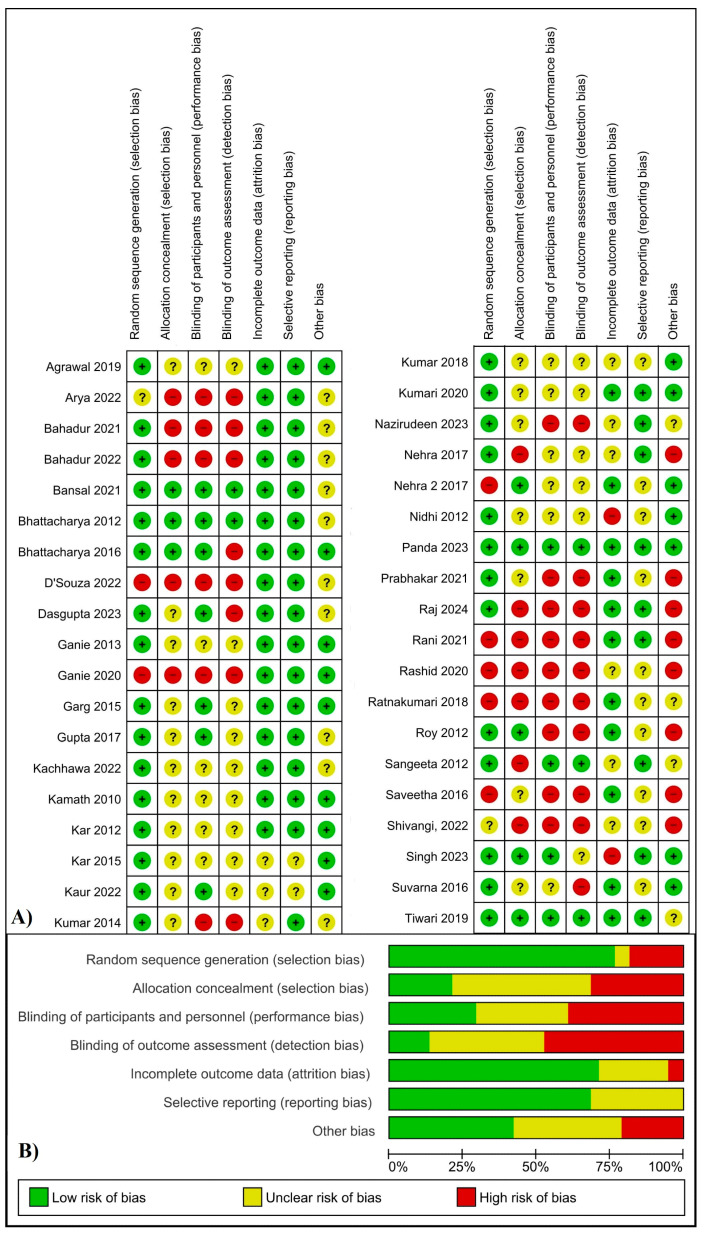
(**A**) An overview of risk of bias assessment for individual studies included in the systematic review. Cochrane risk-of-bias tool was used to assess the six types of potential bias in included studies, rating each as low bias (+) (green color), high bias (-) (red color), or unclear bias (?) (yellow color). (**B**) Risk of bias summary graph.

Most of the studies included in this systematic review are of either high risk or moderate risk (Figure 5A). Approximately 70% of studies have addressed random sequence generation, attrition bias, and reporting bias. However, in very few studies (15–25%), allocation concealment and blinding of participants, personnel, and outcomes were performed. Also, half of the studies have not addressed other biases (Figure 5B).

## 5. Discussion

PCOS is a multifaceted syndrome with diverse symptoms and underlying causes, making it management-challenging [65]. Considering India’s unique ethnic, genetic, lifestyle, and environment factors, there is a need for region-specific PCOS treatment guidelines. Therefore, we conducted a systematic analysis to evaluate the effectiveness of various currently used interventions for PCOS in India, to identify the existing gaps and to guide future evidence-based research. However, due to high heterogeneity of the included RCTs, in terms of intervention, population, and outcomes, a detailed meta-analysis was feasible only for limited studies, and the results were synthesized narratively. Additionally, we have provided a detailed explanation of each intervention’s mechanism for better understanding.

### 5.1. Pharmacological Treatment

(a) Insulin sensitizers and anti-androgen drugs

Metformin has emerged as a cornerstone in treating PCOS [40]. Metformin is a biguanide which primarily works as an insulin sensitizer. It activates glucose transporters and helps in glucose uptake by peripheral tissues, especially muscle and liver cells, decreasing serum glucose and insulin levels [37]. Lowering insulin levels leads to a subsequent decrease in ovarian androgen production [66,67]. This can help alleviate symptoms such as hirsutism, acne, and irregular menstrual cycles. Other than this, metformin can also act in an insulin-independent mechanism, directly inhibiting androgen synthesis by theca cells and affecting ovarian steroidogenesis [68]. These actions help regulate menstrual cycles and address metabolic abnormalities associated with PCOS. The majority of studies included in this systematic review reported that metformin effectively improves anthropometric parameters, glucose profile, lipid profile, menstrual cyclicity, and pregnancy rate with mild side effects. However, most studies did not take BMI into account. Only one study, which categorized patients based on BMI, stated that metformin is effective only in obese PCOS patients and not effective in non-obese patients. The International Evidence-based Guideline for PCOS 2023 recommended that metformin should be prescribed for anthropometric and metabolic outcomes (insulin resistance, glucose and lipid profiles) in obese PCOS adult women. It can also be considered for adolescent and non-obese PCOS patients, but limited evidence is present for them. It could also be considered for pregnant women to reduce pre-term birth; however, evidence suggests that it is not practical for other pregnancy-related issues like gestational diabetes mellitus (GDM), miscarriages, preeclampsia, birth weight, etc. [69]. Metformin also showed mild adverse effects, such as gastrointestinal side effects, in some cases [70].

Inositol is a dietary supplement available in isoforms such as myo-inositol (MI) and D-chiro-inositol (DCI) that may act as insulin sensitizers. Myo-inositol can act as a structure basis for a variety of secondary metabolites (e.g., PI3 kinase: phosphatidyl inositol 3-kinase, inositol phosphate), that can act as a messenger to improve the function of insulin receptors, and thereby enhancing glucose uptake and reducing insulin resistance, glucose, insulin levels, and androgen level [38,71]. Therefore, it can restore ovarian function and alleviate symptoms such as hirsutism and acne. It is also involved in follicular development and oocyte maturation, enhancing the oocyte quality and resolving fertility issues [72]. Our meta-anlysis results were in alignment with international guidelines that recommended that inositol (in any form) could be considered for improvement in metabolic outcomes because it has fewer adverse effects. However, there is limited evidence for its efficacy in improving hirsutism, ovulation, and weight. In fact, they recommend using metformin over inositol for hirsutism and central adiposity despite metformin’s more GI-adverse effects. However, its use for the treatment of infertility in PCOS patients is uncertain, with limited evidence on efficacy, adverse effects, and safety issues [69].

Another class of insulin sensitizers are thiazolidinediones (TZDs: pioglitazone and rosiglitazone), which are primarily generally used to treat type 2 diabetes by improving insulin sensitivity. Thiazolidinediones are agonists of the peroxisome proliferator-activated receptor gamma (PPAR-γ), a nuclear receptor found in adipose tissue, muscle, and the liver. Activation of PPAR-γ leads to changes in the transcription of genes involved in glucose and lipid metabolism and enhances the body’s sensitivity to insulin. Improved insulin sensitivity helps lower circulating insulin levels, which can reduce hyperinsulinemia-driven androgen production by the ovaries [73]. They also increase glucose uptake in muscle and adipose tissue by enhancing the expression of glucose transporter type 4 (GLUT4) [74]. This improves glucose utilization and lowers blood glucose levels, improving PCOS patients’ metabolic control. Two studies included in this systematic review stated that pioglitazone is more efficient in improving clinical and biochemical outcomes when compared to metformin and spironolactone. Another study revealed that a combination of rosiglitazone and metformin is significantly more efficient in improving anthropometric parameters and glucose, insulin, and HDL levels and a combination of rosiglitazone and spironolactone is more efficient in decreasing testosterone and HDL levels as compared to rosiglitazone alone. A meta-analysis conducted by Melin et al. (2024) involving 13 RCTs stated that metformin should remain the first-line insulin sensitizer for managing body weight and other metabolic issues in adult PCOS patients because it was superior to rosiglitazone or pioglitazone [75], whereas Abdalla et al. (2024) (24 RCTs, 975 subjects) reported that rosiglitazone and pioglitazone increased weight and BMI more significantly than the placebo and metformin. However, rosiglitazone was better than metformin in reducing LH, and pioglitazone was better than the placebo in reducing insulin and TAGs levels [76].

Spironolactone is a medication commonly used as a potassium-sparing diuretic, but due to its anti-androgenic properties, it can also be employed in the treatment of PCOS. Spironolactone inhibits key enzymes (17α-hydroxylase and 17,20-lyase) involved in androgen synthesis, blocks the binding of androgens to androgen receptors, and increases sex hormone-binding globulin (SHBG) levels, reducing overall androgen production and bioactive free testosterone. Thereby, it may help in alleviating symptoms of hyperandrogenism in PCOS, such as hirsutism, acne, alopecia, and menstrual irregularities [77,78]. One RCT in our study indicated that combination of metformin and spironolactone was better for improving menstrual regularities, FG score, testosterone, and glucose and insulin levels as compared to either drug alone. However, another study revealed that spironolactone is more effective in overweight PCOS patients as compared to lean PCOS patients. Meta-analysis of 24 RCTs by Bashir et al. (2023) concluded that spironolactone could not improve hirsutism and other PCOS symptoms more statically significantly than finasteride, flutamide, and metformin [79]. However, Zeng et al. (2023) reported that a combination of metformin and spironolactone was more efficient in decreasing BMI and androgen levels as compared to metformin alone, but with no significant effect on FG score and no additional side effects [80]. Other anti-androgens that are being tested for PCOS management are cyproterone acetate, flutamide, finasteride, and bicalutamide. International guidelines and Alesi et al. (2023) stated that anti-androgens could be considered to treat hirsutism in PCOS patients only if the use of OCPs is not tolerated, contraindicated, or showing a sub-optimal response after a minimum period of 6 months [69,81].

NAC is a potent antioxidant because it gets converted into metabolites that stimulate glutathione production. It can act as an insulin sensitizer due to its activity on pancreatic insulin secretion. It also protects insulin receptors from oxidative damage, likely improving glucose levels and preventing hyperinsulinemia-induced insulin resistance [39,40]. It has been studied for its potential benefits in PCOS treatment, although its use has yet to be universally accepted or established in clinical guidelines. Two studies in this review concluded that NAC could improve clinical, biochemical, and hormonal parameters in PCOS patients with almost similar efficacy to metformin but with fewer side effects. Therefore, it can be considered a substitute for insulin sensitizers in managing PCOS. Meta-analysis of 11 RCTs (869 patients) conducted by Liu et al. (2023) revealed that NAC decreased metabolic parameters such as BMI, weight, fasting blood glucose, fasting insulin, LDL, cholesterol, and triglycerides more than metformin [82]. Another meta-analysis involving 15 RCTs (2330 patients) stated that NAC did not significantly improve fertility outcomes such as ovulation rate, pregnancy rate, and miscarriage rate when compared to metformin [83]. However, a meta-analysis of 18 RCTs (2185 patients) conducted by Asl et al. (2023) reported that NAC was effective in reducing testosterone levels and increasing FSH and estrogen levels but ineffective for changing endometrial thickness, number of follicles, LH, progesterone, and SHBG levels [84]. NAC is a promising drug for PCOS treatment; however, more research is needed to establish its effectiveness for metabolic and reproductive outcomes.

(b) Anti-obesity drugs

Obesity and PCOS are closely linked, with obesity exacerbating many of the symptoms and complications associated with PCOS, making weight management a critical component of treatment. Although active lifestyle modifications such as restricted diet and exercise are the primary interventions to control weight, if needed, anti-obesity drugs such as glucagon-like-peptide-1 (GLP-1) receptor agonist (liraglutide, semaglutide, exenatide) and orlistat are being prescribed internationally for weight management in adult PCOS patients. Orlistat works by inhibiting the enzyme pancreatic lipase, which breaks down dietary fats into smaller molecules the body can absorb. By blocking this enzyme, orlistat reduces fat absorption by about 30%, leading to a decrease in calorie intake and promoting weight loss [85]. Two RCTs included in this systematic review stated that both metformin and orlistat can be used for weight management in PCOS patients with fewer side effects in the case of orlistat. Graff et al. (2016) reported that orlistat was superior to metformin in reducing body weight and BMI but equivalent in changing HOMA-IR, testosterone, and insulin in obese/overweight PCOS patients [86]. Wang et al. stated that liraglutide was superior to metformin and orlistat in decreasing body weight and waist circumference [87]. Goldberg (2024) reported that liraglutide, semaglutide, and orlistat were superior to the placebo for improving anthropometric outcomes [88].

(c) Oral contraceptive pills (OCPs)

OCPs have been the first-line medication for concurrent treatment of menstrual irregularity, acne, and hirsutism [89]. They also improve endometriosis-related pelvic discomfort, prevent menstrual migraines, treat dysmenorrhea and menorrhagia, and lower the risk of ovarian and endometrial cancer [90]. Ethinyl estradiol is a synthetic form of estrogen used in hormonal contraceptives and hormone replacement therapies. It is frequently combined with progestins in a variety of oral contraceptives. Drospirenone, gestodene, and desogestrel are synthetic progestins commonly used as oral contraceptives [20]. Drospirenone has anti-androgenic and anti-mineralocorticoid properties. Gestodene and desogestrel are third-generation progestins. Studies from our review have shown that ethinylestradiol with gestodene results in a significant decrease in triglyceride, cholesterol, and LDL levels [45]. Recent findings in the meta-analysis (eight RCTs) have shown that compared to OCP alone, the combined treatment of OCP (COCP) and orlistat was more successful in enhancing the ovulation, pregnancy, and overall effective rates, as well as decreasing the weight, hormonal, lipid, and insulin metabolic profiles in PCOS women [91]. Another meta-analysis (19 RCTs) finding showed that combined regimens containing an anti-androgen (EE/cyproterone acetate) may reduce hyperandrogenism more effectively than conventional COCPs; however, they increased the risk of venous thrombotic events [92]. Problems in PCOS women may occasionally get worse due to side effects of COCPs, such as weight gain, mood swings, and adverse effects on cardiometabolic risk factors [93].

The international guidelines recommended the use of COCPs for the management of hirsutism and menstrual regularity in PCOS women of the reproductive-aged group. They recommended that using high-dose (>30 µg) and low-dose (<30 µg) ethinylestradiol does not show any significant differences in the treatment of hirsutism in PCOS women. Further, compared to other COCPs, the 35 μg ethinyl estradiol with cyproterone acetate formulations should be used as a second-line therapy since they balance the advantages and side effects, including the risk of venous thromboembolism. Additionally, when prescribing COCPs to adults and adolescents with PCOS, the relative and absolute contraindications, as well as the adverse effects of COCPs, must be taken into account and discussed individually. The choice of COCP should be individualized, considering the patient’s comorbidities and potential contraindications.

(d) Ovulation induction drugs

Letrozole and CC are the two most commonly used ovulation induction drugs (six RCTs) in India for PCOS treatment. Letrozole acts as an aromatase inhibitor that blocks estrogen biosynthetic pathways. It has been used in the application of a wide range of infertility cases, including PCOS [94]. Aromatase is a microsomal member of the cytochrome P450 hemoprotein-containing enzyme complex superfamily that synthesizes estrogens by catalyzing three consecutive hydroxylation reactions [95]. Letrozole leads to low levels of estrogens, which increases FSH levels and further growth and maturation of ovarian follicles, leading to ovulation. Our one included study has shown that letrozole can significantly increase ovulation, follicular development rate, and progesterone compared to the placebo in clomiphene citrate-resistance PCOS patients. CC has both estrogenic-agonist and estrogenic-antagonist properties; it binds to estrogen receptors in the hypothalamus (competitive binding to estrogen receptors) and blocks the negative feedback of estrogen, which leads to an increase in gonadotropin-releasing hormone (GnRH) levels. GnRH stimulates the pituitary to release FSH and LH, further stimulating the ovary and leading to ovulation [96]. A further study included in our systematic showed that treatment with a combination of CC and metformin results in a significantly higher ovulation rate as compared to CC alone in PCOS women. In the meta-analysis, letrozole was slightly more effective than CC for increasing the pregnancy rate. Similar findings have been observed in studies by Abu Zaid (2024) and Liu (2023), where letrozole is better than CC in endometrial thickness, monofollicular development, ovulation and pregnancy rates, and live-birth rates [97,98].

International evidence-based guidelines have shown/recommended that letrozole ought to be the first-choice pharmaceutical treatment for ovulation induction in infertile anovulatory women with PCOS with no other infertility factors. Further, in some countries, letrozole is off-label, and clinicians can use CC for OI. Moreover, guidelines also suggest that a combination of CC with metformin is better than using metformin or CC alone in PCOS women for treatment of anovulation and to improve live-birth rates.

### 5.2. Non-Pharmacological Treatment

(a) Lifestyle modification (physical exercise and yoga)

Non-pharmacological treatments play a crucial role in managing polycystic ovary syndrome (PCOS), offering a holistic approach that addresses the root causes and symptoms of the condition without relying solely on medication. These treatments focus on lifestyle modifications, which can significantly improve symptoms and overall health. Diet and exercise are fundamental components of non-pharmacological management [99,100]. Adopting a balanced diet rich in whole grains, proteins, fruits, vegetables, and healthy fats helps to regulate blood sugar levels and improve insulin sensitivity [101,102]. Regular physical activity, including aerobic exercises like walking or cycling, strength training, and yoga, can significantly enhance insulin sensitivity, promote weight loss, and improve cardiovascular health [103,104]. We found only one RCT with a very small sample size of 15 patients per group that compared the efficacy of multi-modular intervention with regular pharmacological treatment. They demonstrated that adding lifestyle intervention with regular medical treatment provided a holistic approach and improved clinical and anthropometric outcomes more efficiently. Two other studies revealed that yoga significantly improves anthropometric, biochemical, hormonal, and reproductive outcomes compared to conventional physical exercise. International guidelines on PCOS also recognize lifestyle intervention as a core focus for PCOS management and recommend the use of it for improving metabolic health issues such as central adiposity and lipid profile and optimizing the overall health and quality of life of patients [69]. However, there is no definitive evidence supporting one specific diet composition or exercise over another for health outcomes in PCOS women. A sustainable, healthy eating habit tailored to individual preferences and goals should be promoted, and overly restrictive or nutritionally unbalanced diets should be avoided. Multiple other studies also suggested the same about lifestyle interventions and that they must be prescribed with or without pharmacological interventions [99,105,106].

(b) Supplements such as vitamin D (Vit D)

Additionally, the use of certain herbal and nutritional supplements like vitamins (B9, B12, D, E, K), vitamin-like nutrients (α-lipoic acid and bioflavonoids), minerals (Ca, Zn, Se, Cr) and other formulations (omega-3 fatty acids, probiotics, melatonin, cinnamon, etc.) can complement dietary and lifestyle changes [107]. These supplements have shown promise in improving insulin sensitivity, reducing inflammation, and supporting metabolic health. While these supplements should be used under the guidance of a healthcare provider, they can offer additional benefits beyond what lifestyle changes alone can achieve. Two out of three RCTs included in this review stated that vitamin D supplementation improves the biochemical outcomes of PCOS women. Other international studies stated that vitamin D supplementation could regularize the menstrual cycle, improve pregnancy and ovulation rates, and balance the androgen and other hormone levels [108,109].

(c) Probiotics and herbal treatment

We also found one RCT for each probiotic and herbal treatment (furocyst), and both were effective in improving PCOS symptoms compared to the placebo. Tabrizi et al. (2022) stated that probiotic supplementation may lead to improvement in some of the anthropometric (weight and BMI) and biochemical outcomes (glucose and insulin levels, lipid profile, testosterone, etc.) in PCOS women [110]. Lakshmi et al. (2023) described various herbal medicines that can be beneficial in PCOS and need to be tested. They suggest the possibility of use of *Vitex agnus-castus* and *Curcuma longa* for ovulatory cycle regulation, *Glycyrrhiza glabra*, *Linum usitatissimum*, *Mentha spicata*, *Cocus nucifera*, and *Punica granatum* for anti-androgen properties, *Cinnamomum cassia* and *Aloe vera* to restore glucose sensitivity and estrus cyclicity, *Foeniculum vulgare*, *Panax ginseng*, *Cimicifuga racemosa*, *Pimpinella anisum* L, and *Trigonella foenumgraecum* to reduce LH and FSH levels, and *Zingiber officinalis* and *Tribulus terrestris* for ovulation induction [111]. Manouchehri (2023) showed that herbal compounds such as aloe vera, chamomile, *Vitex agnus-castus*, liquorice, ginseng, cinnamon, *Stachys lavandulifolia*, and fennel are helpful for the treatment of various symptoms of PCOS [112].

## 6. Limitations in the Current Landscape of Research for PCOS Management in India

In this systematic review, we included 38 studies, encompassing all the RCTs conducted on Indian PCOS patients since 2010. However, we have observed several shortcomings in the current research landscape that hinder a comprehensive understanding of PCOS management in India. These are as follows:

➢ *Heterogeneity in study designs and interventions*: The included studies employed a wide range of interventions targeting different outcomes, with significant variability in participant characteristics, drug dosages, and treatment durations. This high heterogeneity complicates result interpretation and limits direct comparisons;
➢ *Small sample sizes*: Most of the studies had small sample sizes, with only two studies enrolling 100 participants per group. This restricts the statistical power and robustness of the meta-analysis findings;
➢ *Lack of multi-center trials*: Most studies are single-center trials, which reduce the generalizability of the results to the broader population;
➢ *High risk of bias*: Maximum studies exhibited a high risk of bias, affecting the external validity of their results;
➢ *Short-term follow-up*: All the included studies assessed outcomes over only 3–12 months. Given that PCOS is a chronic condition, long-term studies are crucial to evaluate the sustainability of treatments and their impact on the overall health of women.

These limitations highlight the need for large-scale, multi-centric trials with standardized protocols, adequately powered sample sizes, and rigorous methodologies. They should also account for confounding factors and prioritize long-term outcome assessments to provide more robust evidence for PCOS management in the Indian context.

## 7. Future Perspective

This systematic review has identified significant gaps in current PCOS management research, uncovering several underexplored areas. For instance, many studies on metformin and other drugs have not accounted for BMI despite its potential influence on treatment outcomes in obese versus non-obese PCOS patients. Additionally, there is a notable lack of research on the effects of metformin during pregnancy in women with PCOS. The use of inositol for infertility treatment in PCOS patients remains uncertain, with limited evidence on its efficacy, safety, and adverse effects. Furthermore, the effectiveness of TZDs varies between Indian and international studies, signaling the need for further investigation. Data on the impact of anti-obesity agents (such as orlistat, rimonabant, and sibutramine) on various outcomes, particularly reproductive outcomes in women with PCOS, are also scarce, making this a priority for future research. Several pharmacological treatments, including anti-androgens (e.g., cyproterone acetate, flutamide, finasteride, and bicalutamide), sodium glucose co-transporter 2 (SGLT2 inhibitors), and statins, are still underexplored. Emerging therapies, such as miRNA therapy, glucagon receptor antagonists, and imeglemin, should also be explored.

Non-pharmacological approaches, which target the underlying causes of PCOS and often have fewer side effects than pharmacological treatments, are equally important. However, research on these methods—including yoga, physical exercise, herbal treatments, dietary supplements, and probiotics—is limited in India and requires further investigation. For exercise and yoga, future trials could determine the optimal daily duration and specific types of activities that most effectively alleviate PCOS symptoms. At present, there is no high-quality evidence supporting the efficacy and safety of nutritional supplements, probiotics, and herbal medicines for women with PCOS. High-quality trials are urgently needed to evaluate the effectiveness and safety of these interventions.

## 8. Conclusions

PCOS is an endocrine disorder that affects a large proportion of women globally. Its multifactorial nature and varied clinical presentation across different life stages necessitate a multidisciplinary approach to diagnosis and management. This review critically evaluated the diverse treatment modalities including lifestyle interventions, pharmacological options like insulin sensitizers, OCPs, etc. and highlighted their use, efficacy, side effects, and limitations. Evidence suggested that pharmacological drugs provide symptomatic relief, while lifestyle modifications can provide consistent long-term benefits in general. However, for PCOS treatment the strategy must be designed according to the patient’s symptoms and need, which may be challenging, particularly in the Indian context, where diverse sociocultural factors influence healthcare access and compliance. Our findings identify the significant gaps in interventions being used as well as identify the lacunae in the designing of clinical trials. This will guide future population-based research with a robust protocol to optimize patient outcomes and inform evidence-based practice in India.

## Figures and Tables

**Table 1:** Characteristics of 38 included studies.

	Study and Citation	Study Design	Diagnostic Criteria for PCOS	PCOS Women Characteristics	Location	Interventions	N (Finally Analyzed Patient)	Duration of Intervention (Months)	Outcomes Measured	Outcome in Which Significant Difference Observed (Better-Performing Group)	Reference
	Pharmacological Treatment										
	Insulin Sensitizers										
1	Raj et al., 2024	Open-label RCT	Rotterdam 2003	Age: 18–40 years	SRM Medical College Hospital and Research Centre, Kattankulathur, Tamil Nadu, Chengalpattu, India	1 = metformin (500 mg), TDS; 2 = myo-inositol (1 g/day)	1 = 36, 2 = 35	3 months	MC, P, FBS, FI, L, HDL, LDL, C, TAGs, FSH, LH, LH/FSH	P (50% vs. 26.6%), HDL (1), side effects (1 > 2)	[27]
2	Nazirudeen et al., 2023	RCT	Rotterdam 2003	Age: 18–35 years, BMI > 23 kg/m^2^	Government Rajaji Hospital, Madurai, Tamil Nadu, India	1 = metformin (500 mg), BD; 2 = metformin (500 mg) + myo-inositol (1.1 g) + D-chiro-inositol (27.6 mg), BD	1 = 27, 2 = 26	6 months	MC, APs, FBS, FI, IR, L, T, SHBG, LH, FSH, AMH, USG, P, QoL	MC (2)	[28]
3	Arya et al., 2022	RCT	Rotterdam 2003	Age: 18 to 37 years	M.G.M. Medical College, Kishanganj, Bihar, India	1 = metformin (500 mg), TDS; 2 = N acetyl cysteine (600 mg), TDS	1 = 50, 2 =50	6 months	APs, MC, FG, T, TSH, Pr, LH, FSH, FBS, FI, USG	BMI, WHR, T (2)	[29]
4	Bahadur et al., 2021	RCT	Rotterdam 2003	Age: 18 to 45 years	AIIMS Rishikesh, Uttrakhand, India	1 = metformin (500 mg), BD; 2 = metformin (500 mg) + myo-inositol (550 mg) + D-chiro-inositol (150 mg)	1 = 36, 2 = 36	6 months	MC, FG, APs, LH/FSH, LH, FSH, T, DHEAS, HDL, LDL, TAGs, C, PI, FI, IR	A, MC, LH/FSH, HDL, LDL, C, PI (2)	[30]
5	Prabhakar et al., 2021	RCT	Rotterdam 2003	Age: 20–38 years, infertile, BMI < 30 kg/m^2^	AIIMS, New Delhi, India	1 = metformin (500 mg), TDS + myo-inositol (2 g), BD; 2 = myo-inositol (2 g), BD; both groups: after 3 months, three cycles of ovulation induction (letrozole, 2.5 mg, day 2–6 of menstrual cycle) + intrauterine insemination	1 = 57, 2 = 59	6 months	BMI, L, FBS, FI, IR, LH, FSH, TSH, Pr, T, SHBG	P (45.5% vs. 42%, *p* > 0.05) (2 > 1), side effects (1 > 2)	[31]
6	Kumari et al., 2020	Prospective study, randomly assigned	Rotterdam 2003	Age: 18 to 37 years	NSMCH, Bihta, Patna, Bihar, India	1 = metformin (500 mg), TDS; 2 = N acetyl cysteine (600 mg), TDS	1 = 50, 2 = 50	6 months	APs, MC, FG, T, TSH, Pr, LH, FSH, FBS, FI, USG	FG, FI (1 = 2), BMI, WHR (0.08), T (0.23 nmol/L) (2 > 1)	[32]
7	Rashid et al., 2020	Open-label RCT	Rotterdam 2003	Age: 18–40 years	Sher-i-Kashmir Institute of Medical Sciences (SKIMS), Srinagar, J&K, India	1 = spironolactone (50 mg) + Vit D 4000 IU; 2 = metformin (1000 mg) + Vit D 4000 IU; 3 = pioglitazone (30 mg) + Vit D 4000 IU OD	1 = 30, 2 = 30, 3 = 30	6 months	MC, FG, FBS, IR, FI, T, C, TAGs, LDL, HDL	MC, LDL, Vit D, Ca (1 = 2 = 3), W (1.66 kg), FG (2.5), FBS (7.04 mg/dL), FI (4.99 mg/dL), IR (1.13), HDL, LH, leptin (2.97 ng/mL) (3 > 2 and 1: *p* < 0.05)	[33]
8	Ganie et al., 2020	Prospective non-randomized study	Rotterdam 2003	NM *	Sher-i-Kashmir Institute of Medical Sciences, Srinagar, J&K, India	1 = rosiglitazone (4 mg), OD; 2 = rosiglitazone (4 mg), OD + spironolactone (50 mg), OD; 3 = rosiglitazone (4 mg), OD + metformin (500 mg), BD	1 = 30, 2 = 40, 3 = 34	6 months	APs, FG, LH, FSH, T, C, TAGs, C, IR, FBS, PI, MC	MC, FG, IR, PI (1 = 2 = 3), T (30.21 ng/dL) (2 > 3 and 1: *p* < 0.05), W (2.26 kg), BMI (1.01 kg/m^2^), WHR (0.03), FBS (9.97 mg/dL), FI (10.1 mg/dL) (3 > 2 and 1: *p* < 0.05)	[34]
9	Agrawal et al., 2019	RCT	Rotterdam 2003	Age: 20–38 years, infertile, BMI < 30 kg/m^2^	AIIMS, New Delhi, India	1 = metformin (500 mg) + myo-inositol (600 mg), TDS; 2 = metformin (500 mg), TDS; both groups: after 3 months, three cycles of ovulation induction (letrozole, 2.5 mg, day 2–6 of menstrual cycle) + intrauterine insemination	1 = 60, 2 = 60	6 months	LBR, MC, P, OI, OHS, BMI, FG, A, LH, FSH, T, SHBG, AMH, FBS, FI, IR, C, LDL, HDL	MC, P, IR, LBR (1)	[35]
10	Tiwari et al., 2018	RCT	Rotterdam 2003	NM *	Dr. Baba Saheb Ambedkar Medical College, New Delhi, India	1 = fixed exercise + placebo; 2 = fixed exercise + metformin (850 mg), BD	1 = 33, 2 = 33	6 months	MC, W, BMI, W:H, WC, FG, T, TAGs, C, GTT	MC (55.17% vs. 83.33%), W (1.08 kg vs. 2.5 kg), WC (2.56 cm vs. 4.75 cm), W:H (0.02 vs. 0.04) (2 > 1: *p* < 0.05)	[36]
11	Kumar et al., 2018	RCT	NM *	Age: 18 to 37 years, Married	SRM Medical College Hospital and Research Centre, Tamil Nadu, India	1 = metformin (500 mg), TDS; 2 = N acetyl cysteine (600 mg), TDS	1 = 50, 2 = 50	3 months	FBS, FI, G:I, LH, FSH	FBS, FI, G:I, LH, FSH (1 = 2), side effect (1 > 2)	[37]
12	Nehra at al., 2017	Open-label RCT	Androgen Excess Society 2006	Age: 15 to 45 years, Married	PGIMS, Rohtak, Haryana, India	1 = myo-inositol (1 g), BD 2 = metformin (500 mg), TDS	1 = 30, 2 = 30	6 months	FG, FI, G:I, IR, L, LH, FSH, T	G:I, IR, L, LH, FSH, T (1 = 2)	[38]
13	Nehra at al., 2017	Open-label RCT	Androgen Excess Society 2006	Age: 15 to 45 years, Married	PGIMS, Rohtak, Haryana, India	1 = myo-inositol (1 g), BD; 2 = metformin (500 mg), TDS	1 = 30, 2 = 30	6 months	W, BMI, WHR	W, BMI, WHR (1 = 2)	[39]
14	Ganie et al., 2013	Open-label RCT	Androgen Excess Society 2006	NM *	Sher-i-Kashmir Institute of Medical Sciences (SKIMS), Srinagar, J&K, India	1 = metformin (1000 mg/d); 2 = spironolactone (50 mg/d); 3 = metformin (1000 mg/d) + spironolactone (50 mg/d)	1 = 56, 2 = 51, 3 = 62	6 months	MC, FG, BMI, APs, BP, LH, FSH, T, FBS, FI, IR	MC (6.13 ± 2.54 to 11.86 ± 3.20 cycles/y), FG (4.02), T (1.52 nmol/L), FBS (6.16 mg/dL), FI (7.6 µIU/mL) (3 > 2, 3 > 1: *p* = 0.01)	[40]
15	Sangeeta et al., 2012	Double-blind RCT	Rotterdam 2003	Age:18–30 years	Gandhi Hospital, Hyderabad, Andhra Pradesh, India	1 = metformin (500 mg), BD; 2 = pioglitazone (15 mg) OD	1 = 43, 2 = 42	6 months	MC, FG, HDL, VLDL, C, PI, IR, SHBG, FAI, LH, FSH	MC, FG, OR (1 = 2), C (6.4% vs. 20.67%), HDl (17% vs. 64%), VLDL (15% vs. 32%), IR (15% vs. 55%), SHBG (21% vs. 80%), FAI (22.6% vs. 47%), L/H ratio (0.045 vs. 0.736), OI (2 > 1 (*p* < 0.05)	[41]
	**Anti-obesity drugs**										
16	Rani et al., 2021	Prospective clinical trial	Presence of chronic anovulation, one or more signs of clinical hyperandrogenism, and/or endocrinological abnormalities	Age: 18–32 years, treatment-naïve	Medinirai Medical College and Hospital, Palamu, Jharkhand, India	Spironolactone (100 mg/day) + food restriction (1400 kCal/day); 1 = lean (BMI < 24.5 kg/m^2^);2 = overweight (BMI > 25 kg/m^2^)	1 = 13, 2 = 12	12 months	FG, BP, BMI, TAGs, C, HDL, FBS, GTT, I, IR	TA (−22%, *p* < 0.05), G, IR (2 > 1), HDL (24%, *p* < 0.05) (1 > 2), FG (1 = 2)	[42]
17	Saveetha et al., 2016	Prospective study	PCOS diagnosed by USG and irregular cycles and anovulation	Age: 18 to 45 years	ESI Hospital, Ayanavaram, Chennai, Tamil Nadu, India	Metformin (500 mg), BD; 1 = obese (BMI > 29.9); 2 = non-obese (BMI = 18–24.5)	1 = 37, 2 = 53	12 months	BP, BMI, FBS, PBS, C	BMI (1.41 kg/m^2^, *p* = 0.005), W (3.37 kg, *p* = 0.04), BP (6.6 mmHg, p 0.032), FBS (5.4 mg/dL, *p* = 0.037), PBS (5.44 mg/dl, *p* = 0.042), C (6.38 mm/dL, *p* = 0.047) (1 > 2), not significant effect for 2	[43]
18	Kumar et al., 2014	RCT	Rotterdam 2003	Age: <40 years, BMI > 23 kg/m^2^, infertile	Manipal Assisted Reproduction Center, Kasturba Medical College, Manipal University, Manipal Karnataka, India	1 = metformin (stepwise, max to 500 mg TDS) + fertility fitness program (diet, exercise, and lifestyle intervention); 2 = orlistat (120 mg BD) + fertility fitness program (diet, exercise, and lifestyle intervention); 3 = fertility fitness program (diet, exercise, and lifestyle intervention)	1 = 30, 2 = 30, 3 = 30	3 months	MC, FG, A, APs, LH, FSH, DHEAS, SHBG, FAI, FI, FBS, HOMA-IR, HDL, LDL, C, TAGs, UAG	APs, W (1 = 2), side effects (1 > 2)	[44]
	**OCPs**										
19	Dasgupta et al., 2024	RCT	Rotterdam 2003	Non-obese, reproductive age	Rampurhat Government Medical College and Hospital, Birbhum, West Bengal, India	1 = ethinylestradiol 20 µg + drosperinone 3 mg; 2 = ethinylestradiol 15 µg + gestodene 60 µg; 3 = ethinylestradiol 30 µg + desogestrel 150 µg	1 = 51, 2 = 51, 3 = 51	1 year	BMI, FG, TSH, C, TC, HDL, TC/HDL, LDL, IR, T	TAGs (1.60 mg/dL), C (10.14 mg/dL), LDL (8.76 mg/dL) (2 > 1 and 3) (*p* = 0.01), TSH (1.6 mg/dL; *p* = 0.01) (1 > 2 and 3), T (1.38 ng/mL, *p* = 0.00), IR (0.28, *p* = 0.003) (3 > 1 and 2)	[45]
20	Kachhawa et al., 2022	Open-label RCT	Rotterdam 2003	Age: 15–24 years	AIIMS, New Delhi, India	1 = myo-inositol (500 mg) and D-chiro-inositol (150 mg), BD; 2 = ethinyl estradiol (20 µg) + drospirenone (3 mg), OD	1 = 33, 2 = 34	6 months	BMI, WHR, AMH, LH, FSH, T, IR, MC, FI	FI (3.82 µU/m, *p* = 0.05), IR (0.82, *p* < 0.05) (1 > 2), LH (1.17, mIU/mL), T (0.04, ng/mL), AMH (2.21 ng/mL), MC (27.27% vs. 100%) (2 > 1)	[46]
21	Shivangi et al., 2022	RCT	Rotterdam 2003	PCOS	PGIMS, Rohtak, Haryana, India	1 = combined oral contraceptives (COCPs); 2 = cyperoterone acetate + ethinyl estradiol	1 = 50, 2 = 50	6 months	BP, HDL, TAGs, FBS	Glucose level deranged in both groups (1 > 2), HDL, TAGs deranged in both groups (1 < 2)	[47]
22	Suvarna et al., 2016	Prospective study, physician’s discretion	Rotterdam 2003	Age: 18 to 45 years	M.S. Ramaiah Medical College and Hospitals, Karnataka, India	1 = metformin (1 g) BD 2 = OCPs (drospirenone 3 mg + ethinyl estradiol 30 μg), OD, on days 1–21 of the menstrual cycle	1 = 11, 2 = 11	6 months	MC, BMI, T, DHEA	MC (100% and 72%) (2 > 1), BMI (2.2 kg/m^2^ vs. 2.11 kg/m^2^), T (9.66 vs. 6.85 ng/dL), DHEA (31.71 vs. 30.8 mcg/dL) (1 = 2)	[48]
23	Bhattacharya et al., 2016	RCT	Rotterdam 2003	PCOS	S.C. Das Memorial Medical and Research Centre, Kolkata, India	1 = drospirenone (3 mg) + ethinyl estradiol (30 μg), on days 1–21 of the menstrual cycle with no treatment on days 22–28 (21 + 7 regim); 2 = drospirenone (3 mg) + ethinyl estradiol (20 μg) (24 + 4 regimen)	1 = 46, 2 = 48	6 and 12 months	SHBG, A, BMI, APs, FG, FAI, T, PBS, PI, PBS:PI	SHBG(2) at 6 and 12 months, PBS:PI (2) at 6 months	[49]
24	Bhattacharya et al., 2012	RCT	Androgen Excess Society 2006	Age: 18–35 years	S.C. Das Memorial Medical and Research Centre, Kolkata, India,	1 = desogestrel (30/150 mg); 2 = cyproterone acetate (35/2000 mg); 3 = drospirenone (30/3000 mg)21 days followed by a 7-day gap, cyclically	1 = 49, 2 = 51, 3 = 53	12 months	FG, SHBG, FAI,	FG (−5.29 vs.−1.69 vs. −2.12), SHBG (142.91 vs. 99.53 vs. 131.52 nmol/L), FAI (10.57 vs. 5.58 vs. 7.89) (2 > 3 > 1)	[50]
	**Ovulation Induction Drugs**										
25	Panda et al., 2023	Triple-blind RCT	Rotterdam 2003	Infertile, age: 20–35 years	AIIMS, Mangalagiri, Andhra Pradesh, India	1 = letrozole (2.5 mg) + placebo; 2 = letrozole (2.5 mg) + clomiphene citrate (50 mg), OD, 3–7 days of menstrual cycle	1 = 40, 2 = 40	1 treatment cycle	OI, P	OI (73% vs. 38%; *p* = 0.003) (2 > 1)	[51]
26	Bansal et al., 2021	Assessor-masked RCT	Rotterdam 2003	Age: 18–35 years, anovulatory infertility	AIIMS, Jodhpur, Rajasthan, India	1 = letrozole (2.5 mg/day), OD, 2–6 days of menstrual cycle, stepwise increase to 7.5 mg/day in subsequent menstrual cycles; 2 = clomiphene citrate (50 mg/day) 2–6 days of menstrual cycle, stepwise increase to 150 mg/day in subsequent menstrual cycles	1 = 41, 2 =39	3 cycles or until conception	ET, OI, MF, P, DT	P, DT, MF, OI (1)	[52]
27	Kar et al., 2015	RCT	Rotterdam 2003	Infertility, treatment-naïve	Kar Clinic and Hospital Pvt. Ltd., Bhubaneswar, Odisha, India	1 = clomiphene citrate (50 mg/day) 2–6 days of menstrual cycle, stepwise increase to 150 mg/day in subsequent menstrual cycles; 2= metformin (850 mg/day increase to 1700 mg/day after 2 weeks); 3 = CC (50–150 mg/day) + metformin (850–1700 mg/day)	1 = 32, 2 = 24, 3 = 24	6 months or until CC-resistant	P, LBR, OI, M, MF	LBR (1 < 2 < 3), MF (2), OI (27.1%: *p* = 0.03) (3 > 2 and 1)	[53]
28	Roy KK et al., 2012	Prospective RCT	Rotterdam 2003	Age: 20–35 years	AIIMS, New Delhi, India	1= letrozole (2.5–5 mg); 2 = clomiphene citrate (50–100 mg);orally from days 3–7 of menstrual cycle	1 = 98, 2 = 106	3 treatment cycles	OR, ET, P, LBR	ET, P, LBR (2)	[54]
29	Kar et al., 2012	RCT	Rotterdam 2003	Infertility, treatment-naïve	Kar Clinic and Hospital Pvt. Ltd., Bhubaneswar, Odisha, India	1 = clomiphene citrate (100 mg/day) 2–6 days of menstrual cycle, stepwise increase to 150 mg/day in subsequent menstrual cycles; 2 = letrozole (2.5 mg/day)	1 = 51, 2 = 52	1 treatment cycle	OR, ET, MF, OI, P, M	MF, P (2)	[55]
30	Kamath et al., 2010	RCT	Rotterdam 2003	CC resistance (200 mg)	Reproductive Medicine Unit, Christian Medical College, Vellore, Tamilnadu, India	1 = letrozole, 2.5 mg, OD, 2–6 days of the menstrual cycle; 2 = placebo	1 = 17, 2 = 17	1 treatment cycle	FDR, Pro, ET, BMI, FSH, BR, P, OR, MF	FDR (27% vs. 0%; *p* = 0.015), Pro (24.42 vs. 1.66 nmol/L; *p* = 0.014), OR (33.3% vs. 0%; *p* = 0.006) (1 > 2)	[56]
	**Lifestyle Modification**										
31	D’Souza et al., 2022	Purposive sampling, prospective intervention study	NM *	Age: 18–30 years	Father Muller College of Nursing, Mangaluru, Karnataka, India	1 = regular medical treatment + diet + walk + exercise; (a) insulin resistance diet (healthy fat and protein, few carbohydrates), prescribed by dietician; (b) brisk walk (30 min) in first month, jogging from next month (30 min); (c) core muscle exercise (half push-ups and burpees), 20 min/day, 5 days/week; 2 = regular medical treatment	1 = 15, 2 = 15	6 months	APs, BMI, WHR, FG, A, FBS, MC, QoL	WHR (z-value = 3.328, *p* < 0.001), FG (z-value 2.296, *p* < 0.022), A, QoL (1)	[57]
	**Yoga**										
32	Ratnakumari et al., 2018	Single-blinded prospective study, pre–post clinical trial	Rotterdam 2003	Age: 18–35 years	Government Yoga and Naturopathy Medical College, Arumbakkam, Chennai, Tamil Nadu, India	1 = yoga (asanas, pranayama, relaxation technique, kriyas, etc.) 20 min + naturopathy (hydrotherapy, mud therapy, massage therapy, fasting, and natural diet), 6 days/week, excluding menstrual days; 2 = control	1 = 22, 2 = 22	3 months	USG, APs, MC	PCOM, APs (1)	[58]
33	Nidhi et al., 2012	RCT	Rotterdam 2003	Age: 15–18 years	Residential school in Anantpur, Andhra Pradesh, India	1 = group lecture, Surya Namaskara, prone asanas, standing asanas, supine asanas, sitting asanas, guided relaxation, breathing techniques, OM meditation; 2 = group lecture, brisk walk, standing exercises, supine exercises, sitting exercises, supine rest, normal breathing	1 = 35, 2 = 36	12 weeks	FI, FBS, IR, HDL, C, BMI, WC, HC	FI (−1.30 ± 4.65 vs. 1.60 ± 8.19 pmol/L; *p* < 0.05), FBS (−4.26 ± 6.97 vs. 0.64 ± 7.94 mmlo/L; *p* < 0.01), IR (0.38 ± 0.92 vs. 0.29 ± 1.56; *p* < 0.05), TAGs (12.94 ± 10.72 vs. 6.44 ± 10.80 mmol/L; *p* < 0.05), HDL, LDL (8.20 ± 9.83 vs. 2.85 ± 15.14 mmol/L; *p* < 0.05), VLDL (2.40 ± 1.97 vs. 1.34 ± 2.23 mmol/L; *p* < 0.05), C (9.37 ± 11.30 vs. 2.86 ± 17.75 mmol/L; *p* < 0.05) (2 > 1)	[59]
	**Vit D Supplement**										
34	Bahadur et al., 2022	RCT	NM	Age 20–35 years, insulin resistance (HOMA-IR > 2.5), Vit D < 20 ng/mL, BMI < 30 kg/m^2^	AIIMS, Rishikesh, Uttarakhand, India	1 = metformin (500 mg), BD + Vit D3 (1000 IU/day); 2 = metformin (500 mg), BD + Vit D3 (4000 IU/day)	1 = 36, 2 = 36	3 months	APs, FG, A, FBS, FI, PI, L, LH, FSH, T, DHEAS, IR, MC	FG (1.19 vs. 2.81; *p* = 0.027), PBS (32.89. 49.03; *p* = 0.005), FI (6.87 vs. 12.44, *p* = 0.040), MC, IR (1.79 vs. 4.38; *p* = 0.002) (2 > 1), MC (25% and 27.81%), A (2.42 and 2.81) (1 = 2)	[60]
35	Gupta et al., 2017	Double-blind RCT	Rotterdam 2003	Age: 18–45 years	ESI PGIMSR Basaidarapur, New Delhi, India.	1 = Vit D (60,000 IU/week); 2 = placebo	1 = 25, 2 = 25	3 months	LH, FSH, Pr, Cor, Est, T, TSH, FI, Vit D, Ca, FBS, PBS, C, TAGs, HDL, C, IR	FBS (5.88 mg/dL; *p* = 0.041), IR (1.38; *p* = 0.003), FI (5.34 mg/dL; *p* = 0.021), HDL (26.34 mg/dL; *p* = 0.035), QUICKI (0.024; *p* = 0.001), MC (20% to 48%) (1 > 2)	[61]
36	Garg et al., 2015	Double-blind RCT	Rotterdam 2003	Age: 18–35 years	AIIMS, New Delhi, India	1 = metformin (1500 mg/day) + Vit D (4000 IU/day) (given once a month 120,000 IU oral dose); 2 = metformin (1500 mg/day) + placebo	1 = 15, 2 = 17	6 months	IR, G, DI, I, BP, L,	No significant change	[62]
	**Probiotics**										
37	Kaur et al., 2022	Double-blind RCT	Rotterdam 2003	Age: 18–40 years	PGIMER, Chandigarh, India	1 = Multi-strain probiotic oral capsule (10 billion CFU) (2 months OD then 4 months BD) (Lactobacillus acidophilus UBLA-34, L. rhamnosus UBLR-58, L. reuteri UBLRu-87 (each of 2 billion CFU); L. plantarum UBLP-40, L. casei UBLC-42, L. fermentum UBLF-31, Bifidobacterium bifidum UBBB-55 (each of 1 billion CFU); and fructo-oligosaccharides (100 mg)); 2 = placebo; both groups: dietary (carbohydrate (55–60%), fat (20%), and proteins (20–25%)) + exercise (two sets of exercise plans (phase I and II, 3 months each)	1 = 48, 2 = 49	6 months	USG, T, DHEAS, I, FBS, LH, FSH, HOMA-IR, W, WHR, BMI, QoL	MC (18.8% vs. 6.1%; *p* = 0.23), T (0.3 vs. 0.29 nmol/L; *p* = 0.043), WHR (0.02 vs. 0.01; *p* = 0.027), WC (3.9 vs. 2.3 cm; *p* = 0.030), QoL (*p* = 0.034) (1)	[63]
	**Herbal Treatment**										
38	Singh et al., 2023	Double-blind RCT	Rotterdam 2003	Age: 18–45 years, BMI < 42 kg/m^2^	PGIMER, Chandigarh, India	1 = placebo; 2 = furocyst (500 mg capsule), BD (fenugreek; Trigonella foenum graecum extract); both groups: convential health diet with carbohydrate (40–50%), fat (30%), and protein (20–25%)	1 = 95, 2 = 113	3 months	FBS, FI, SGOT, U, Cr, HDL, LDL, TAGs, LH, FSH, TSH, Pr, USG, SHBG, T, MC, IR, GTT,	FBS (16.63 mmol/L, *p* = 0.001), LH (26.4%, *p* < 0.01), FSH (21.1%, *p* < 0.01), TSH (0.47 mU/L, *p* = 0.01), Pr (2.51 ng/mL, *p* = 0.017), MC (41.15%), FG (27.1%), Cy [37.3% (right), 38.2% (left)], G, IR, C (35.76 mg/dL, *p* = 0.001), LDL (18.05 mg/dL, *p* = 0.001), TAGs (20.6 mg/dL, *p* = 0.015), T (0.38 ng/dL, *p* = 0.001) (2 > 1)	[64]

* NM = not mentioned. Abbreviations: A: global acne score, ALP: alkaline phosphatase, AMH: anti-Mullerian hormone, APs: anthropometric parameters, BD: twice a day, BMI: body mass index, BP: blood pressure, C: total cholesterol, Ca: free calcium, CFU: colony-forming units, Cr: serum creatinine, DHEAS: dehydroepi-androsterone sulfate, DT: duration of treatment, ET: endometrial thickness, FAI: Free Androgen Index, FBS: fasting glucose, FDR: follicular development rate, FG: modified Ferriman–Gallwey score, FI: fasting insulin, FSH: follicle-stimulating hormone, GTT: glucose tolerance test, HDL: high-density lipoprotein, I: insulin, IR: homeostatic model assessment for insulin resistance (HOMO-IR), L: fasting lipid profile, LBR: live-birth rate, LDL: low-density lipoprotein, LH: luteinizing hormone, M: miscarriage, MC: menstrual cycle regularity, MF: mature follicle, MF: monofollicular development, OD: once a day, OI: ovulation induction, OR: ovulation rate, P: pregnancy, PBS: postprandial glucose, PCOM: polycystic ovary morphology, PCOS: polycystic ovarian syndrome, PI: postprandialinsulin, Pr: prolactin, Pro: progesterone, QoL: quality of life, SGOT: serum glutamic oxloacetic transaminase, SHBG: sex hormone-binding globulin, T: serum testosterone, TAGs: triglycerides, TDS: thrice a day, TSH: thyroid-stimulating hormone, U: serum urea, USG: pelvic ultrasound, Vit D: vitamin D, W: reduction in weight, WC: waist circumference, WHR: waist-to-hip ratio.

## Data Availability

Attached as Supplementary File.

## Supplementary Materials

The following supporting information can be downloaded at https://www.mdpi.com/article/10.3390/ph18050680/s1: Search results of databases; PRISMA checklist. Reference [113] is cited in the Supplementary Materials.

## Abbreviations

A: global acne score, ALP: alkaline phosphatase, AMH: anti-Mullerian hormone, APs: anthropometric parameters, BD: twice a day, BMI: body mass index, BP: blood pressure, C: total cholesterol, Ca: free calcium, CFU: colony-forming units, Cr: serum creatinine, DCI: D-chiro-inositol, DHEAS: dehydroepiandrosterone sulfate, DT: duration of treatment, ET: endometrial thickness, FAI: Free Androgen Index, FBS: fasting glucose, FDR: follicular development rate, FG: modified Ferriman–Gallwey score, FI: fasting insulin, FSH: follicle-stimulating hormone, GI: gastrointestinal, GTT: glucose tolerance test, HDL: high-density lipoprotein, I: insulin, HOMO-IR: homeostatic model assessment for insulin resistance, L: fasting lipid profile, LBR: live-birth rate, LDL: low-density lipoprotein, LH: luteinizing hormone, M: miscarriage, MC: menstrual cycle regularity, MF: mature follicle, MF: monofollicular development, OD: once a day, OI: ovulation induction, OR: ovulation rate, P: pregnancy, PBS: postprandial glucose, PCOM: polycystic ovary morphology, PCOS: polycystic ovarian syndrome, PI: postprandial insulin, Pr: prolactin, Pro: progesterone, QoL: quality of life, SGLT2: sodium glucose co-transporter 2, SGOT: serum glutamic oxloacetic transaminase, SHBG: sex hormone-binding globulin, T: serum testosterone, TAGs: triglycerides, TDS: thrice a day, TSH: thyroid-stimulating hormone, U: serum urea, USG: pelvic ultrasound, Vit D: vitamin D, W: reduction in weight, WC: waist circumference, WHR: waist-to-hip ratio.
